# Implementing AI in healthcare—the relevance of trust: a scoping review

**DOI:** 10.3389/frhs.2023.1211150

**Published:** 2023-08-24

**Authors:** Emilie Steerling, Elin Siira, Per Nilsen, Petra Svedberg, Jens Nygren

**Affiliations:** ^1^ School of Health and Welfare, Halmstad University, Halmstad, Sweden; ^2^ Department of Health, Medicine and Caring Sciences, Linköping University, Linköping, Sweden

**Keywords:** trust, artificial intelligence, implementation, healthcare, scoping review

## Abstract

**Background:**

The process of translation of AI and its potential benefits into practice in healthcare services has been slow in spite of its rapid development. Trust in AI in relation to implementation processes is an important aspect. Without a clear understanding, the development of effective implementation strategies will not be possible, nor will AI advance despite the significant investments and possibilities.

**Objective:**

This study aimed to explore the scientific literature regarding how trust in AI in relation to implementation in healthcare is conceptualized and what influences trust in AI in relation to implementation in healthcare.

**Methods:**

This scoping review included five scientific databases. These were searched to identify publications related to the study aims. Articles were included if they were published in English, after 2012, and peer-reviewed. Two independent reviewers conducted an abstract and full-text review, as well as carrying out a thematic analysis with an inductive approach to address the study aims. The review was reported in accordance with the PRISMA-ScR guidelines.

**Results:**

A total of eight studies were included in the final review. We found that trust was conceptualized in different ways. Most empirical studies had an individual perspective where trust was directed toward the technology's capability. Two studies focused on trust as relational between people in the context of the AI application rather than as having trust in the technology itself. Trust was also understood by its determinants and as having a mediating role, positioned between characteristics and AI use. The thematic analysis yielded three themes: individual characteristics, AI characteristics and contextual characteristics, which influence trust in AI in relation to implementation in healthcare.

**Conclusions:**

Findings showed that the conceptualization of trust in AI differed between the studies, as well as which determinants they accounted for as influencing trust. Few studies looked beyond individual characteristics and AI characteristics. Future empirical research addressing trust in AI in relation to implementation in healthcare should have a more holistic view of the concept to be able to manage the many challenges, uncertainties, and perceived risks.

## 1. Introduction

Artificial intelligence (AI) can be understood as “a computerized system that is equipped with the capacity to perform tasks or reasoning processes that we usually associated with the intelligence level of a human being” (1). These systems have the potential to transform healthcare at many levels and solve many of its current challenges (2–4), e.g., by reducing costs and workloads, improving efficiency and quality, as well as by making earlier and more accurate diagnoses (2, 5). The expectations on AI are high and the European Union (2) and the European Commission are making significant investments in AI (6).

Despite the rapid development of AI and its potential benefits when implemented in healthcare, the process of translation into practice has been slow (7). AI systems tend to be complex, unpredictable, lack evidence, and difficult to grasp, hence the many uncertainties and risks related to its use, e.g., patient harm, bias, and lack of privacy (2). Trust in AI and its trustworthiness have therefore been regarded as important aspects to address (6, 8, 9). Based on literature from other scientific fields, trust is fundamental for a functioning health system (10) where patients are in vulnerable situations since it is known to increase the tolerance of uncertainty, as well as to reduce the perceived complexity (11). Trust is understood as a way of dealing with uncertainty (12), and according to Luhmann (13), trust is an attitude which leaves room for risk-taking behavior. To be trustworthy is a characteristic of someone who is competent to perform an action and has the moral attitude toward those who depend on the performance (14, 15). Being trustworthy helps in gaining trust but does not imply trust *per se* (16, 17).

Most research in AI in healthcare has so far been primarily focused on AI's performance (18), fairness, trustworthiness (8, 19–22), legal and ethical issues (21–27), and transparency and explainability (19–22, 24, 27).

Aspects such as AI's influence and interaction with the context in which it is implemented are also important to consider for successful implementation of AI (28). There appears to be a general lack of empirical research investigating implementation processes in relation to AI in healthcare (7, 28, 29). Health professionals are trusted and authorized to give advice and treatment based on their profession and expertise (30–33), and an implementation of AI into practice is believed to disrupt healthcare by questioning these health professionals' existing authority, as well as influencing organizational structures, roles, and practices (1, 7, 29). The many challenges, uncertainties, and perceived risks reflect the importance of trust in AI in relation to implementation in healthcare.

In order to successfully implement AI into routine applications in healthcare and change clinical practice, an understanding of trust in AI in relation to the change processes is needed. No previous studies exploring the concept trust in AI in relation to implementation in healthcare have to our knowledge been performed, which implies there could be a lack of conceptual clarity. Without a clear understanding of trust in AI, it could be difficult to identify implementation strategies, which means that AI will not advance despite the significant investments and possibilities. The aim of this paper was thus to explore the scientific literature regarding how trust in AI is conceptualized in relation to implementation in healthcare and what influences trust in AI in relation to implementation in healthcare.

## 2. Methods

### 2.1. Study design

We chose a scoping review methodology to explore all relevant literature addressing trust in AI in relation to implementation in healthcare, since this methodology is useful for identifying knowledge gaps, scoping a body of literature, or clarifying concepts (34). We used the methodological framework developed by Arksey and O'Malley (35) and followed the five stages: (1) identifying the research question, (2) identifying relevant articles, (3) selecting articles, (4) charting the data, and (5) collating, summarizing, and reporting the results. The review followed the recommendations in the Preferred Reporting Items for Systematic Reviews and Meta-Analysis for Scoping Reviews (PRISMA-ScR) checklist (34), and since it was based on publicly available studies there was no ethical consideration related to the handling of personal and sensitive information. A review protocol based on Arksey and O'Malley's (35) framework was developed, and the final version of the protocol can be found in Data Sheet 1.

### 2.2. Identifying the research question

To address the aim, we formulated two research questions:

1. How is trust in AI conceptualized in relation to implementation in healthcare?
2. What influences trust in AI in relation to implementation in healthcare?

### 2.3. Identifying relevant articles

A thorough search for published literature was developed and carried out together with an experienced librarian. Search terms included a combination of terms related to implementation, AI, and healthcare. We used standardized subject headings describing the terms and subcategories provided by the databases. Truncation of words allowed for alternative endings and were used for implementation, improvement, innovation, and intervention. The term trust had to be specific since the aim was to explore how trust was conceptualized in AI in relation to implementation in healthcare. The electronic database search was recorded in a table (Data Sheet 2). An initial search was carried out in CINAHL and PubMed to identify keywords and subject headings, which were then included in the search strategy for the selected databases. Five electronic databases (PubMed, CINAHL, PsychINFO, Web of Science and Scopus) were systematically searched to identify relevant scientific literature. In addition, reference lists of the identified research articles were reviewed manually.

The eligibility criteria ensured that the content of the included studies was relevant to the research question (36). The focus was on trust in AI in relation to implementation in healthcare, and there was no restriction placed on the type of methodology used in the paper (e.g., qualitative, quantitative, mixed methods or theoretical). To be included, articles had to: (a) address “trust” in AI in (b) relation to implementation in healthcare. Although there are closely related terms for trust, we found it important to be specific since the aim was to conceptualize “trust” in AI in relation to its implementation in healthcare. Articles were excluded if they were non-English, not available in full text, not peer reviewed or published before 2012 (Table 1). The decision to exclude articles published before 2012 was made to allow a focus on more recent development of AI, due to its fast-changing nature. AI was uncommon in healthcare settings prior to 2012 (3).

**Table 1:** Inclusion and exclusion criteria.

Inclusion criteria	Exclusion criteria
- Studies addressing trust in relation to implementation of AI in healthcare. - Peer reviewed	- Abstract missing. - Published before 2012. - Not in English. - Only mentioning trust.

We defined implementation as “An intentional effort designed to change or adapt or uptake interventions into routines”, which was based on a definition used by two earlier reviews with a focus on implementation of AI into healthcare practice (7, 28). We also made a distinction between trust and trustworthiness, and we excluded studies that were only mentioning trust without giving it further attention or dealing with it in relation to implementation in healthcare.

### 2.4. Selecting articles

The eligible articles were uploaded into Endnote X9 software where duplicates were removed, and thereafter imported into Rayyan. The initial screening of titles and abstracts was conducted in collaboration between two reviewers (authors 1 and 2), who communicated and met regularly to discuss any disagreements or uncertainties regarding which articles to include or exclude based on selected criteria. If agreement could not be reached, the other authors were consulted through discussions. The full article was read if focus of an article was unclear based on title and abstract. In the next step, the same two reviewers (authors 1 and 2) independently conducted the full-text review on the remaining articles, and disagreements and uncertainties were again resolved through discussion with the other authors.

### 2.5. Charting the data

First, we developed a standard data charting form, following the guidelines by Arksey and O'Malley (35), based on characteristics of the articles: (1) country; (2) publication year; (3) methodological design; (4) healthcare setting; (5) aim of the study; (6) application area; (7) intended user; (8) definition of trust (Table 2). Two reviewers (authors 1 and 2) extracted the data from the articles and thereafter confirmed with the other authors. The aim was to explore all relevant literature rather than provide a quantitative or qualitative synthesis. The methodological quality or risk of bias of the included studies were therefore not reviewed, which is consistent with guidance on the conduct of scoping reviews (35, 37).

**Table 2:** Characteristics of included studies .

Author(s)	Country of origin	Methodological design	Healthcare setting	Aim of the study	Application area	Intended user	Definition of Trust
Datta Burton et al. (38), 2021	The United Kingdom	Opinion paper, with empirical support	Neurology	To explore questions of trust between patients and clinicians and between clinicians and researchers.	Brain modelling	Clinicians (unspecified)	A triangle of trust; “relationships between patients and clinicians, and between clinicians and researchers” (38).
Choi et al. (39), 2020	The United States & Canada	Opinion paper, without empirical support	Radiology	To outline several ethical and practical concerns in integrating AI with human cognition in the real-world: bias and pitfalls of AI, ethics of trust and risk regarding AI, and design of the human—AI interface.	Image recognition	Clinicians (radiologist)	“A human's propensity to submit to vulnerability and unpredictability, and nevertheless to use that automation, as measured by intention expressed in speech or writing, or by measurable bodily actions to actually use the automation” (40).
Esmaeilzadeh et al. (41), 2021	The United States	Quantitative: survey study	Healthcare, general	To examine how potential users perceive the benefits, risks, and use of AI clinical applications for their healthcare purposes and how their perception may be different if faced with three healthcare service encounter scenarios.	Diagnosis and treatment	Patients (with acute or chronic conditions)	“Trust can be defined as trust in clinicians and the clinical tools they use (such as AI clinical applications)” (42).
Fan et al. (43), 2018	China	Quantitative: survey study	Hospital	To explore the adoption of artificial intelligence-based medical diagnosis support system by integrating Unified theory of user acceptance of technology and trust theory.	Diagnosis	Clinicians (unspecified)	“The beliefs about a technology's capability rather than its will or its motives.” (44).
Liu & Tao, (45), 2022	China	Quantitative: survey study	Healthcare service delivery	To examine the roles of trust and three AI-specific in public acceptance of smart healthcare services based on an extended Technology Acceptance Model.	Smart healthcare services	The general population	“The degree to which an individual perceives that smart healthcare services are dependable, reliable, and trustworthy in supporting one's healthcare activities” (45).
Prakash & Das, (46), 2021	India	Mixed methods	Radiology	To develop and test a model based on theories of Unified Theory of Acceptance and Use of Technology, status quo bias, and technology trust.	Diagnosis	Clinicians (radiologist)	“The willingness of a party to be vulnerable to the actions of another party…” (47).
Roski et al. (48), 2021	The United States	Opinion paper, without empirical support	Healthcare, general	To describe how AI risk mitigation practices could be promulgated through strengthened industry self-governance, specifically through certification and accreditation of AI development and implementation organizations.	AI, general	N/a	N/a
Yakar et al. (49), 2021	Netherlands	Quantitative: survey study	Radiology, dermatology, and robotic surgery	To investigate the general population's view AI in medicine with specific emphasis on three areas that have experienced major progress in AI research in the past years, namely radiology, robotic surgery, and dermatology.	Diagnosis, communication, and surgery	The general population	N/a

### 2.6. Collating, summarizing, and reporting the results

We then used a thematic analysis with an inductive approach to analyze data associated with the research questions, how trust in AI is conceptualized in relation to implementation in healthcare and what influences trust in AI in relation to implementation in healthcare. We followed the guide of Braun and Clarke (50) with six phases: (1) data familiarization; (2) initial code generation; (3) generating themes; (4) theme review; (5) theme defining and naming; (6) and report production. The first step involved reading and rereading the articles, as well as making notes. Two reviewers (authors 1 and 2) reflected individually and generated independently lists of codes from words and phrases, which were coded regarding trust in AI in relation to implementation in healthcare. The reviewers then compared their codes and interpretations, and the relationships between the codes were discussed, which were referred to as subthemes. The conceptualization of trust was either clearly defined or defined by its determinants. The subthemes were then analyzed, and three overarching themes were generated. All authors discussed continuously the data analysis to enhance its quality and validity. No qualitative data analysis software was used.

## 3. Results

**Figure 1:**
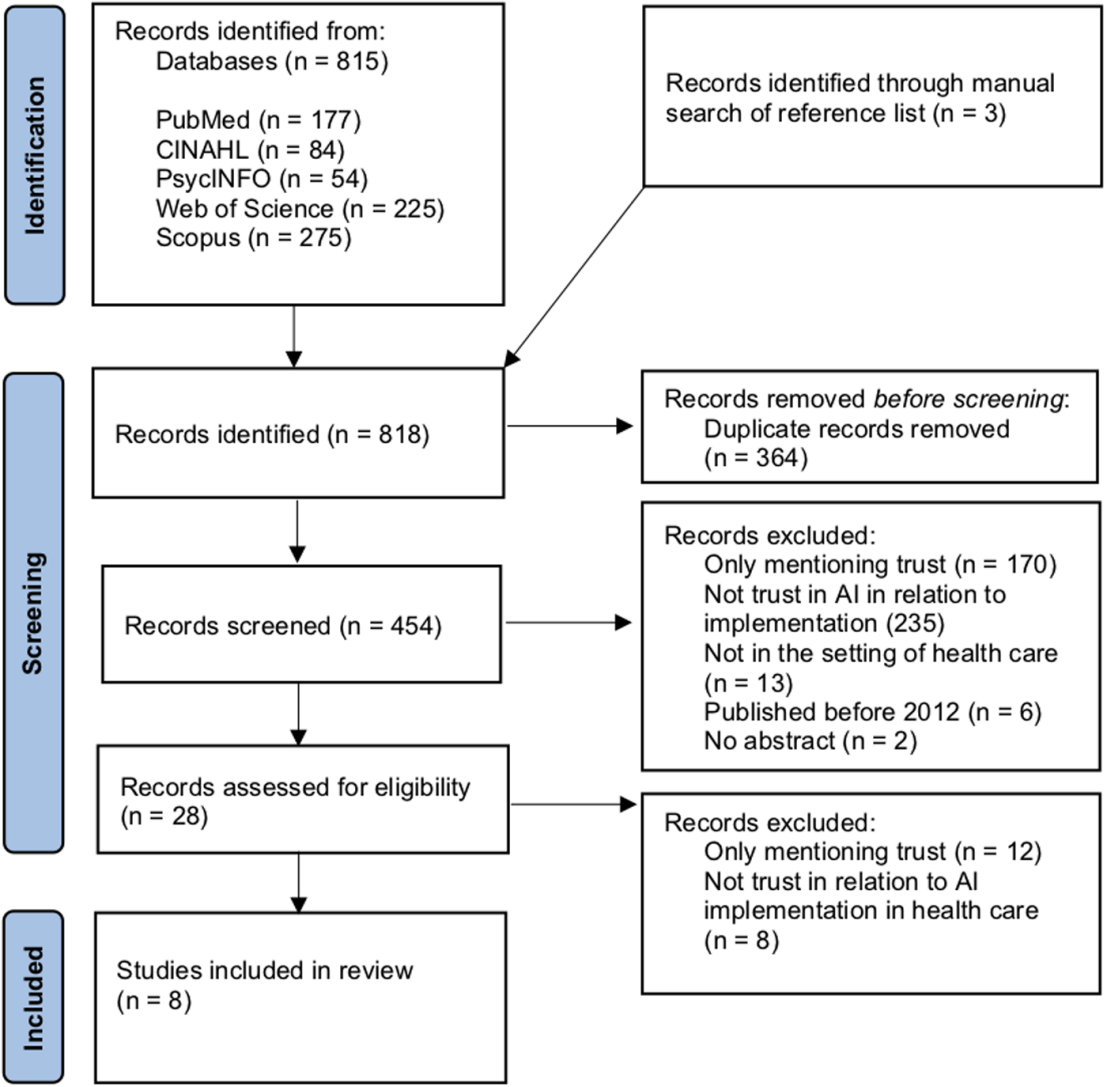
PRISMA-ScR flowchart.

A total of 815 articles were retrieved from the five databases. Three articles were identified through manual searches of reference lists. The number of articles for review was reduced to 454 after duplicates were removed. 426 of the 454 (93.8%) were excluded in the title and abstract screening, for reasons highlighted in Figure 1. The term trust was often only mentioned, but not further addressed (*n =* 170). 235 articles investigated trust but not in AI in relation to implementation, thirteen articles were not in the healthcare setting, six articles were published before 2012 and two articles had no abstract. This resulted in a high number of excluded articles. Only 28 articles remained for full text review. Twelve of these articles were excluded because they only mentioned trust and did not further address or elaborate on the concept in the full text, and eight articles were excluded because they did not address trust in relation to AI implementation in healthcare. A total of eight articles met all criteria and were included in the study.

### 3.1. Study characteristics

The included studies were published between 2018 and 2022. Most articles were from the United States (*n* = 3), two from China, and the remainder from the United Kingdom (*n* = 1), India (*n* = 1), Canada (*n* = 1) and Netherlands (*n* = 1). Many of the studies (*n* = 6) were conducted in hospital settings (neurological practice, radiology, dermatology, and robotic surgery), except for two studies which involved healthcare management at home and healthcare in general. AI was often used for diagnosis (*n* = 4). Other application areas were brain modelling (1), image recognition (1), smart healthcare services (1), treatment (1), surgery (1), communication (1). One study was too general to have a specific purpose. Four studies were based on quantitative studies, three were opinion papers, and one mixed method. The studies examined the perceptions of different intended users: clinicians (*n* = 4), general population (*n = 2*), and patients (*n* = 1). The characteristics of the included studies are shown in Table 2.

### 3.2. How is trust in AI conceptualized in relation to implementation in healthcare?

Six out of the eight studies provided a definition of trust (Table 2). Most empirical studies had an individual perspective where trust was directed toward the technology's capability (*n *= 4), e.g., describing trust as human's propensity or willingness to submit to the vulnerability of the technology's capability (39, 43, 46) or the perception of AI as being dependable, reliable, and trustworthy in supporting healthcare activities (45). Two studies had a contextual perspective and focused on trust as relational between people in the context of the AI application rather than having trust in the technology itself. Datta Burton et al. (38) argued that it is necessary to develop the human side of these tools, which represents a triangle of trust relationships: between patients and clinicians, and between clinicians and researchers. Esmaeilzadeh et al. (41) focused on care encounters and understood trust as the degree to which an individual believes that the clinical encounter is trustworthy and referred to Reddy et al. (42) who understood trust as “Trust is in the clinicians and the clinical tools they use”. Two studies only defined trust indirectly by describing trust determinants (48, 49).

### 3.3. What influences trust in AI in relation to implementation in healthcare?

The inductive coding yielded three themes regarding what influences trust in AI implementation in healthcare, which could be understood as interconnected: *individual characteristics, AI characteristics, and contextual characteristics*. These themes were based on 10 subthemes and 34 codes (Table 3).

**Table 3:** Influences of trust in relation to implementation of AI in healthcare based on inductive thematic analysis.

Themes	Subthemes	Codes	Articles
Individual characteristics	Demographic characteristics	Age, education, sex/gender, geographic origin, and employment.	(43, 45, 46, 49)
	Knowledge	Usage experience, tacit knowledge, and tech skills.	(38, 43, 45, 46, 49)
	Personal traits	Cognition and positive attitude.	(43, 46, 49)
	Health condition	Health condition and healthcare consumption.	(41, 49)
AI characteristics	Individualization	Personalization, privacy, and anthropomorphism.	(41, 45)
	“Black box”	Self-learning, non-transparent, and autonomous.	(38, 39, 41, 46, 48)
	Technical objectivity	Data-driven, accurate, lack of moral values, and lack of empathy.	(38, 39, 41, 46, 49)
Contextual characteristics	Healthcare culture	Medical area, task complexity, “skilled clinician”, professional expertise, custodians, and opinion of important others.	(38, 41, 43, 46, 49)
	Interpersonal relations	Collaboration, personal interactions, and mutual understanding	(38, 41, 48, 49)
	Governance	Policies, guidelines, and standards/regulation.	(38, 39, 41, 48)

#### 3.3.1. Individual characteristics

The individual characteristics capture those qualities that make the individuals different from each other, such as age, sex/gender, personality. These characteristics influence individuals’ trust in AI in relation to an implementation in healthcare. *Demographic characteristics* such as gender, age and education were found to relate to trust by moderating the relationship between antecedents and behavioral intention (*n = *4). For example, being male, higher educated, employed or student, and with Western background were predictors of trust in AI among the general population (49). Disposition to trust technology (a person's general tendency to be willing to depend on technology) varied among clinicians based on their living experiences (43) and cultural background (43, 46). *Knowledge* and technological skills were found to influence trust in AI (*n = 5)*, which emphasized the need for education and training (49). Four studies understood trust as influenced by earlier usage experience or technological skills (38, 43, 45, 46), e.g., radiologists were used to highly complex machines in their routine clinical practice, and ease of use may therefore not be a concern in the adoption-related decision making (46). *Personal traits* such as cognition and having a positive attitude were associated with higher levels of trust (*n = 3*), e.g., disposition to trust technology was related to trust in AI use (43, 46), and understood as influenced by the individual's cognition and personality (46). *Health conditions* and healthcare consumption were also something that influenced trust (*n = 2*), e.g., individuals with chronic conditions may not trust AI clinical applications if no physician interaction were included in healthcare delivery (41) and individuals who utilized less healthcare were associated with a higher level of trust in AI (49).

#### 3.3.2. AI characteristics

Trust in relation to the characteristics of AI was frequently mentioned in the literature, where aspects such as its performance, capacity, and trustworthiness were focused on. AI's ability to *individualization* was shown to enhance trust, which was understood as care tailored to the patients' unique needs (*n = 2*). Personalization was based on patients' health information, which required sharing sensitive personal data and caused concerns such as risks of privacy breaches (41, 45). AI's anthropomorphic characteristics enhanced trust in AI in relation to an implementation since it generated a sense of social presence. It was referred to as the perceived level of humanlike characteristics such as human appearance, self-consciousness, and emotion (45). AI characteristics such as “*black box”*, self-learning, non-transparent and autonomous characteristics brought uncertainty and threatened trust in the implementation of AI (*n *= 5), since inputs and operations were not visible to the user. *Technical objectivity*, which included characteristics such as data-driven, accuracy, lack of moral values, and lack of empathy, was also related to trust (*n *= *5)*, since they in some cases could produce results that were more accurate and reliable than those of even the most skilled diagnostician (38).

#### 3.3.3. Contextual characteristics

The theme contextual characteristics concerned the influence on trust in AI in relation to implementation in healthcare regarding the context in which individuals and AI are embedded. The contextual characteristics in relation to implementation of AI in healthcare consisted of the following subthemes: *healthcare culture, interpersonal relationships, and governance. Healthcare culture* included medical area, professional expertise, and opinion of important others (*n = 5*). For example, a “skilled clinician” was considered someone who had embodied tacit knowledge through years of experience in a community of experts (38). Opinion of important others, such as clinicians, colleagues, and seniors, shaped individuals’ initial trust (43, 46). Trust in AI in relation to implementation in healthcare depended also on the medical area, e.g., the perceived risks of using AI in radiology and dermatology compared to robotic surgery (49). *Interpersonal relationship,* collaboration, personal interactions, and mutual understanding were found to influence trust (*n = 4*), especially between different stakeholders (38, 48). Thus, reduced communication in relation to AI implementation was believed to result in less trust among patients (41, 49). Yakar et al. (49) investigated trust in AI in the areas of radiology, surgery and dermatology, and the results showed that those who found personal interactions important had less trust in all three areas. *Governance,* including policies, standards, and guidelines had to be defined to enhance trust in AI (*n* *= 4*). The lack of clear guidelines in medical context was believed to lead to more uncertainties and less trust (41). Roski et al. (48) highlighted the importance of different stakeholder-consented framework and goals to enhance trust, which was also a condition for self-governance. Datta Burton et al. (38) suggested policies that encourage greater clinician engagement in the evaluation of a computational model that would lead to more responsible adoption.

## 4. Discussion

This study was conducted to explore the scientific literature regarding how trust in AI is conceptualized in relation to implementation in healthcare and what influences trust in AI in relation to implementation in healthcare. Only eight studies were found to meet the strict inclusion criteria. The results showed that the conceptualization of trust in AI differed between the studies, as well as what they accounted for as influencing trust. We identified three themes that influenced trust in AI in relation to implementation in healthcare: individual characteristics, AI characteristics and contextual characteristics. Most research focused on the individual characteristics or AI characteristics, and the focus was rarely on the context or implementation processes.

AI in healthcare is a relatively new endeavor but the use of AI has become more common in healthcare setting during the past decade (3). Studies on the implementation of AI in healthcare are therefore fairly new research areas. This could explain the low number of included studies, which all were recently published and mostly from high income countries. Another explanation for the low number could be that trust is rarely mentioned in implementation science frameworks, theories, or models (51). The findings showed that the intended users were often clinicians (38, 39, 43, 46), which also aligns with implementation science where the focus is on clinicians rather than patients. Most of the empirical studies were cross-sectional where questionnaires were used to measure trust as the individual's attitudes and perceptions of AI's capability (41, 43, 45, 49) rather than considering other influencing variables. These studies discussed AI at a general level where the individuals had no or very little experience with practical AI tools, instead of addressing trust where the tools have been implemented and used over longer periods. One should thus be careful in using these perspectives in the development of implementation strategies to avoid building strategies on opinions, perceptions, and potential misconceptions rather than on actual experiences. Moreover, these fairly superficial perspectives on trust in AI in relation to implementation give little insight since they do not consider the context and the underlying values.

The conceptualization of trust in AI in relation to implementation in healthcare differed between the included studies. Some studies focused on individual characteristics and AI characteristics (39, 43, 45, 46, 49), and other studies concentrated on the relations between people (38, 41). Trust in AI in relation to implementation in healthcare did not always have a specific definition. Instead, it was understood indirectly as influenced by different characteristics or determinants, and as having a mediating role, positioned between perceptions of AI characteristics and AI use. These different approaches to trust in AI reveal its complexity and the need of having a holistic understanding of the concept spanning different levels and dimensions.

**Figure 2:**
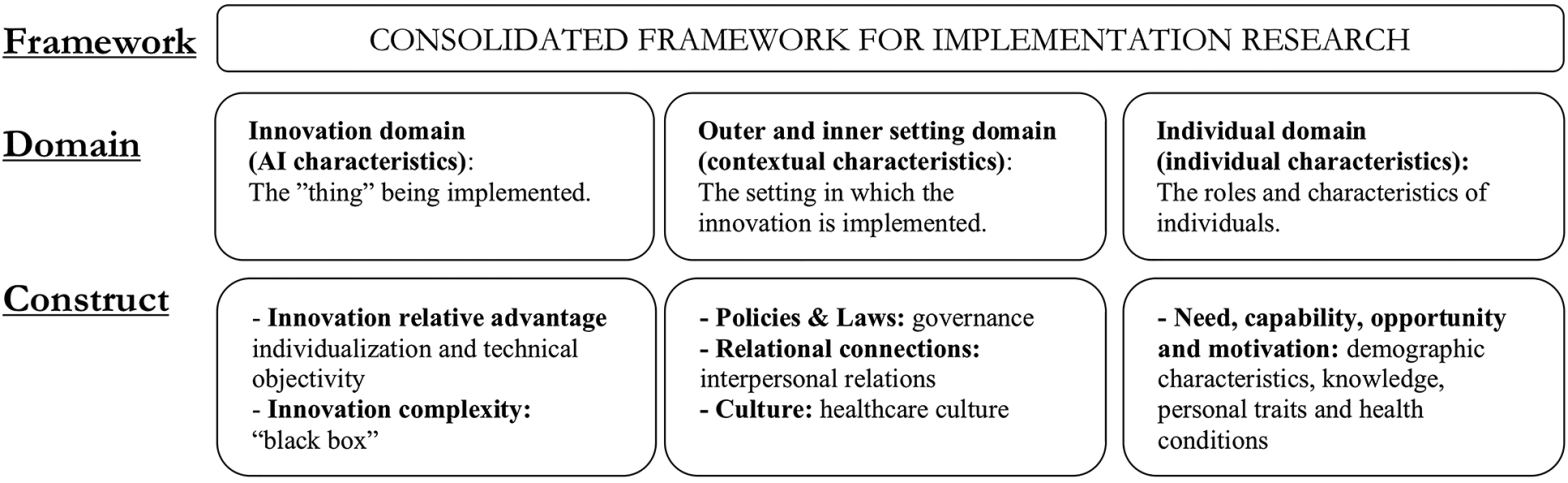
The determinants associated with trust in AI in relation to implementation in healthcare mapped onto CFIR domains and Constructs (55).

The three themes that was found to influence trust in AI in relation to implementation in healthcare can be compared to implementation science, which emphasizes the determinants that influence the implementation by understanding the context in which they are used (52, 53). In line with Leeman et al. (54). The determinants to facilitate implementation need to be known for appropriate strategies to be chosen. The themes are well-aligned with the Consolidated Framework for Implementation Research (CFIR), which is one of the most widely used determinant frameworks in implementation science (Figure 2). Trust could be placed in the assessment category in CFIR, situated between determinants and outcomes, where also the concepts of acceptability, appropriateness, feasibility, implementation readiness and implementation climate are placed (55).

The theme individual characteristics such as an individual's circumstances was shown to influence trust in AI (38, 41, 43, 45, 46, 49). The result showed that individuals in vulnerable positions (less educated people, unemployed, people with non-Western immigration background, older people, and patients with chronic conditions) had low degree of trust in AI (49). Hence, the relationship between trust and the individuals' perception of control or empowerment. This may be consistent with Luhmann (11) who argued that people are willing to trust if they possess inner security. Moreover, perceptions of AI characteristics such as being a non-transparent “black box” with autonomous and self-learning capacity were related to lack of trust in AI since these characteristics obstruct the understanding of its decisions. Knowledge and technological skills were other aspects that were shown to enhance trust in AI, which may also be understood as related to control or empowerment.

This study showed that trust in AI in relation to implementation in healthcare may be related to knowledge within a context. People's perception of AI as meaningful, useful, or valuable contributed to trust (38, 39, 41, 43, 45, 46). The results showed that trust in AI was not only influenced by its “technical” objectivity, efficiency, and accuracy. For example, person-centered care does not only consider medical competence as technical skills but also relational moral competency, empathy, compassion, and trust (41), which could explain why AI's anthropomorphic characteristics and personalization enhanced trust in AI (45). Healthcare culture is based on knowledge within a context and could be why the individuals' trust in AI was often shaped by important others (43, 46, 49), as well as why interpersonal relationships, collaboration and common understanding were found to influence trust (38, 41, 48, 49). It also explains the importance of governance and the need of common guidelines (38, 39, 41, 48).

Knowledge within a context and its influence on trust in AI in relation to implementation in healthcare could be compared to Normalization Process Theory (NPT), another widely used theoretical approach in implementation science. The theory understands implementation as a possible challenge toward individuals' existing ways of working or thinking about care (56). NPT suggests that people need to make sense of AI together to understand their specific roles and responsibilities in relation to AI use in healthcare, and the importance of new agreements and values that give meanings to their actions (57). This could be explained by our ability to contextualize information through narratives (58), which is also in line with Luhmann (11) who viewed trust as possible only in a familiar world.

Only considering AI's technical aspects when implementing AI in healthcare is not enough. AI tools should not be understood apart from the context and the people using them. Existing values and understanding of care can become barriers to trust in AI in relation to implementation in healthcare if there is a lack of coherence. There is thus a need to understand the context in relation to implementation (59) to be able to align AI to existing values (38, 57). Differences in values must be considered for trust to be present when implementing AI in healthcare. The use of AI could thus add value to clinical reasoning rather than competing with it according to Datta Burton et al. (38).

### 4.1. Strength and limitations

The study has some strengths that are worth highlighting. The search was designed together with a librarian and the selection of relevant studies were conducted independently by two reviewers with consensus. We used a comprehensive search strategy and adhered to a structure for scoping reviews outlined by Arksey and O'Malley (35).

The study also has shortcomings that must be considered when interpreting the findings. Trust in AI in relation to implementation in healthcare relates to a young research field, and we found it therefore necessary to include any type of methodology in this study. This means the conceptualization of trust in AI was based on both results and reflections. The study was limited to the published literature in English, and we did not search wider grey literature where we may have identified additional relevant literature. Only a small number of articles met the strict inclusion criteria since many of the articles were excluded because they only mentioned trust or did not address trust in AI in relation to implementation in healthcare. Most of the included studies were conducted in high-income countries and the results may therefore not be relevant to other countries.

### 4.2. Implications and suggestions for future work

This scoping review showed that there were different approaches to trust, which demonstrates that trust can be understood at different levels and dimensions. Only considering one aspect could mean that inappropriate strategies are used to support implementation. For example, there were few empirical studies that addressed trust beyond individual characteristics and AI characteristics. Future empirical studies thus need to have a holistic view on trust. The results also showed that in order to establish trust in AI in relation to implementation in healthcare, it is important to align AI to existing values and to take account of social interactions and negotiants of values in relation to care. This scoping review also found that trust in AI was often influenced by the opinion of important others (43, 46). Future studies could therefore investigate how these important others facilitate trust in AI in relation to implementation in healthcare. Three of the included studies mentioned that trust grows with time and maturity (39, 43, 46). However, none of these studies investigated this change empirically. There is therefore also a need for a better understanding of how trust in AI changes during implementation in healthcare.

## 5. Conclusions

Findings from the scoping review revealed that there is a variation in the scientific literature how trust in AI in relation to its implementation in healthcare has been conceptualized. Trust is often conceptualized by its determinants and having a mediating role, positioned between characteristics and AI use. There were also differences in what was believed to influence trust in AI. We found three themes that influenced trust in AI in relation to implementation in healthcare: individual characteristics, AI characteristics and contextual characteristics. Today, most research focuses only on one or two perspectives, for example the individual characteristics or the AI characteristics. Future studies addressing trust in AI in relation to implementation in healthcare should have a more holistic view on trust to be able to manage the many challenges and develop appropriate strategies to support the implementation of AI in healthcare.

## Data Availability

The original contributions presented in the study are included in the article/Supplementary Material, further inquiries can be directed to the corresponding author/s.
