# Supplementary material for: Implementing AI in healthcare—the relevance of trust: a scoping review
Source: Front Health Serv. 2023 Aug 24;3:1211150. doi: 10.3389/frhs.2023.1211150 (PMC10484529; doi:10.3389/frhs.2023.1211150)
Supplement: Supplementary file 1 [file Datasheet1.docx]

Supplementary Material

**Additional file 1**

**Conceptualizing trust in use of AI in healthcare: a scoping review protocol**

**Introduction**

Artificial intelligence (AI) is believed to disrupt healthcare by transforming the way healthcare is delivered and consumed [1]. AI can learn from big data and perform predefined tasks with high precision without suffering from burnout [2], and as a tool, it has the potential to improve healthcare e.g., by reducing costs and workloads, improving efficiency and quality [3].

On the other hand, there are many uncertainties and risks related to its use. AI is complex, lacks common sense and can make simple mistakes [2]. Also, the “black box” makes it difficult to understand or explain the data [4, 5]. Thus, an implementation of AI into healthcare setting does not only depend on AI’s performance but also on human factors. Trust and trust-building processes are therefore important aspects to consider in relation to an implementation of AI in healthcare. However, there is a lack of deeper understanding of these aspects [5].

To our knowledge, there are no previous studies exploring the concept trust in AI in relation to implementation in healthcare, which implies there could be a lack of conceptual clarity. Therefore, the aim of this study will be to explore the concept trust in AI in relation to implementation, as well as looking at the requirements for building and maintaining trust in the use of AI in healthcare. The results will contribute to deeper understanding of the concept trust in AI in relation to implementation in healthcare, as well as to identify gaps for further research. This will be a first step in the conceptualization of trust in relation to AI implementation in healthcare.

**Methods**

A scoping review method was identified as the most suitable methodology as it is useful for answering broad questions such as “What is known about this concept?”. The methodology is a type of approach used to examine the extent of literature that exist on the topic [6]. To ensure an accurate approach, this scoping review will be guided by Arksey and O’Malley’s [7] framework, following 5 stages: (i) identifying the research question, (ii) identifying relevant studies, (iii) study selection, (iv) retrieving and charting the data, and (v) collating, summarizing, and reporting the results [7].

**Stage 1: Identifying research question**

The focus will be on AI users, and to see how the scientific literature address trust in AI in relation to implementation into the healthcare setting.

The aim will be to explore the concept trust in AI in relation to implementation in healthcare by identifying scientific literature on the subject.

The findings of the study will conceptualize trust in relation to AI use in healthcare. It will also guide researchers by determining gaps and shortcomings in relation to trust when it comes to adoption and use of AI in healthcare setting. This will help facilitating an implementation of AI in healthcare. The study will offer an understanding of how the scientific literature understand and conceptualize trust in AI in relation to implementation in healthcare and what is needed for building and developing trust in AI in healthcare. It will also give guidance to future research.

The proposed scoping review will answer the following questions:

**Review questions:**

1. Identify scientific literature on trust in AI use within healthcare setting.
2. Explore how trust is researched and conceptualized in relation to AI implementation in healthcare.
3. Identify how trust-building could be addressed when implementing AI into healthcare.

**Stage 2: Identifying relevant studies**

A comprehensive search for published literature will be developed and conducted together with an experienced librarian. The aim is to capture a wide literature on the concepts AI, healthcare, implementation and trust. The following databases will be included: Pubmed, CINAHL, PsychINFO, Web of Science Core Collection and Scopus. The search strategy will be piloted to check if keywords and databases are appropriate.

**Stage 3: Study selection**

The eligibility criteria will ensure that the content of the included studies is relevant to the research question [8]. Identifying the search strategy will be an iterative process where a preliminary search will be conducted in CINAHL and PubMed to identify keywords and subject headings, which will be included in the search strategy.

Title and abstract screening will follow the recommendations in the Preferred Reporting Items for Systematic Reviews and Meta-Analysis for Scoping Reviews (PRISMA-ScR) checklist. The result will be scanned for duplicates. There will not be any restriction on methodology used in the paper (qualitative, quantitative, mixed methods or theoretical) [8].

The eligible articles will be uploaded into Endnote X9 software where duplicates will be removed. The included references will then be imported into Rayyan. Between June and August 2022, the reviewers plan to conduct title and abstract screening of all eligible articles. Two independent reviewers will screen the articles by title and abstract, and then at full text, based on the selected exclusion and inclusion criteria. If there are uncertainties whether or not to include an article at title and abstract, the study will be included for full-text review to ensure no articles are excluded without full consideration. The reviewers will meet regularly to discuss any disagreements regarding which articles to include or exclude. A third reviewer will be consulted if agreement cannot be reached. Thereafter, full texts will be screened, and two independent reviewers will analyze these articles. Reference lists of identified articles will be reviewed manually to identify additional relevant studies.

**Stage 4: Charting the data**

A standard data charting form will be developed based on characteristics of the articles, population characteristics and outcomes, following the guidelines by Arksey and O’Malley [7].

**Stage 5: Collating, summarizing and reporting the results**

A narrative report will be included to summarize the extracted data and to complement the results, and how they relate to the research questions and the purpose of the study. The plan is to use a thematic analysis, and follow the guidance of Braun and Clarke [9].

**References:**

Floridi L. Soft ethics, the governance of digital and General Data Protection Regulation. Philos Trans Ser A Math Phys Eng Sci (2020) 22(6):e15154. doi: [10.1098/rsta.2018.0081](https://doi.org/10.1098/rsta.2018.0081).

Asan O, Bayrak E, Choudhury A. Artificial intelligence and Human Trust in Healthcare: Focus on Clinicians. Journal of Medical Internet Research (2020) 22(6):e15154. doi: [10.2196/15154](https://doi.org/10.2196/15154)

Mehta N, Pandit A, Shukla S. Transforming healthcare with big data analytics and artificial intelligence: A systematic mapping study. Journal of Biomedical Informatics (2019) 100:103311. doi: [10.1016/j.jbi.2019.103311](https://doi.org/10.1016/j.jbi.2019.103311).

Topol EJ. High-performance medicine: the convergence of human and artificial intelligence. Nature medicine (2019) 25:44-56. doi: [10.1038/s41591-018-0300-7](https://pubmed.ncbi.nlm.nih.gov/30617339/).

Gille F, Jobin A, Ienca M. What we talk about when we talk about trust: Theory of trust for AI in healthcare. Intelligence-Based Medicine (2020) 1- 3, doi: [10.1016/j.ibmed.2020.100001](https://www.sciencedirect.com/science/article/pii/S2666521220300016)

Tricco AC, Lillie E, Zarin W, O'Brien KK, Colquhoun H, Levac D et al. PRISMA extension for scoping reviews (PRISMA-ScR): checklist and explanation. Annals of Internal Medicine (2018) 169:467–73. doi: [10.7326/M18-0850](https://www.acpjournals.org/doi/10.7326/M18-0850). 

Arksey H, O’Malley L. Scoping studies: Towards a Methodological Framework. International Journal of Social Research Methodology (2005) 8:19-32. doi: [10.1080/1364557032000119616](https://doi.org/10.1080/1364557032000119616).

Booth A, Sutton A, Clowes M, Martyn-St James M. Systematic Approach to a Successful Literature Review. London: Sage Publications (2021). 424 p.

Braun, V. & Clarke, V. (2022). *“Thematic analysis”, in Cooper, H. (Ed.),* APA Handbook of Research Methods in Psychology: Research Designs, American Psychological Association, Washington, DC, Vol. 2, pp. 57-91.
