# Supplementary material for: Implementing AI in healthcare—the relevance of trust: a scoping review
Source: Front Health Serv. 2023 Aug 24;3:1211150. doi: 10.3389/frhs.2023.1211150 (PMC10484529; doi:10.3389/frhs.2023.1211150)
Supplement: Supplementary file 2 [file Datasheet2.docx]

Supplementary Material 2

**Additional file 2**

|  |  | | | | **Pubmed** | | | **CINAHL (Ebsco)** | | | **PsycINFO (ProQuest)** | | | | | **Web of Science Core Collection** | | | **Scopus** | | **Summa** | |
| --- | --- | --- | --- | --- | --- | --- | --- | --- | --- | --- | --- | --- | --- | --- | --- | --- | --- | --- | --- | --- | --- | --- |
| **No.** | **Search block** | | | | 2022-05-31 | | | 2022-05-31 | | | 2022-05-31 | | | | | 2022-05-31 | | | 2022-05-31 | |  | |
| **1** | artificial intelligence in healthcare | | | | 29660 | | | 10 953 | | | 11 776 | | | | | 37 835 | | | 72 217 | |  | |
| **2** | trust | | | | 53375 | | | 46 511 | | | 59 675 | | | | | 126 203 | | | 151 870 | |  | |
| **3** | implementation | | | | 4 057 092 | | | 764 361 | | | 766 316 | | | | | 6 097 764 | | | 7 372 812 | |  | |
| **4** | 1 AND 2 | | | | 270 | | | 150 | | | 161 | | | | | 435 | | | 507 | | **1 523** | |
| **5** | 1 AND 2 AND 3 | | | | 177 | | | 84 | | | 54 | | | | | 225 | | | 275 | | **815** | |
|  | **Total minus duplicates** | | | |  | | |  | | |  | | | | |  | | |  | | **451** | |
|  | Number of duplicates: | | | |  | | |  | | |  | | | | |  | | |  | | **364** | |
|  |  | | | |  | | |  | | |  | | | | |  | | |  | |  | |
|  |  | | | |  | | |  | | |  | | | | |  | | |  | |  | |
|  |  |  | | | |  | | |  | | |  | | | | | |  | |  |  |  |
|  |  | | |  | | |  | | |  | | |  |  |  |  |  |  |  |  |  |  |
|  |  | | |  | | |  | | |  | | |  |  |  |  |  |  |  |  |  |  |
|  | **Search strings** | |  | | | | |  | | |  | | | | |  | | |  | |  | |
|  | Pubmed | | ("neural networks, computer"[MeSH Terms:noexp] OR "artificial intelligence"[MeSH Terms:noexp] OR "deep learning"[MeSH Terms] OR "supervised machine learning"[MeSH Terms] OR "artificial intelligence"[Title/Abstract] OR "deep learning"[Title/Abstract] OR "supervised machine learning"[Title/Abstract]) AND ("delivery of health care"[MeSH Terms] OR "health care"[Title/Abstract] OR healthcare[Title/Abstract] OR medical[Title/Abstract] OR clinical[Title/Abstract]) AND "english"[Language] | | | | | | | | | | | | | | | | | | | |
|  |  | | ("Trust"[MeSh Terms] OR trust[Title/Abstract] OR trusting[Title/Abstract] OR mistrust[Title/Abstract] OR distrust [Title/Abstract] OR antitrust [Title/Abstract] OR entrust [Title/Abstract]) AND (english[Filter]) | | | | | | | | | | | | | | | | | | | |
|  |  | | (implement*[Title/Abstract] OR improv*[Title/Abstract] OR innovat*[Title/Abstract] OR intervent*[Title/Abstract]) AND (english[Filter]) | | | | | | | | | | | | | | | | | | | |
|  |  | | ("neural networks, computer"[MeSH Terms:noexp] OR "artificial intelligence"[MeSH Terms:noexp] OR "deep learning"[MeSH Terms] OR "supervised machine learning"[MeSH Terms] OR "artificial intelligence"[Title/Abstract] OR "deep learning"[Title/Abstract] OR "supervised machine learning"[Title/Abstract]) AND ("delivery of health care"[MeSH Terms] OR "health care"[Title/Abstract] OR healthcare[Title/Abstract] OR medical[Title/Abstract] OR clinical[Title/Abstract]) AND ("Trust"[MeSh Terms:noexp] OR trust[Title/Abstract] OR trusting[Title/Abstract] OR mistrust[Title/Abstract] OR distrust[Title/Abstract] OR antitrust[Title/Abstract] OR entrust[Title/Abstract]) AND "english"[Language] | | | | | | | | | | | | | | | | | | | |
|  |  | | ("neural networks, computer"[MeSH Terms:noexp] OR "artificial intelligence"[MeSH Terms:noexp] OR "deep learning"[MeSH Terms] OR "supervised machine learning"[MeSH Terms] OR "artificial intelligence"[Title/Abstract] OR "deep learning"[Title/Abstract] OR "supervised machine learning"[Title/Abstract]) AND ("delivery of health care"[MeSH Terms] OR "health care"[Title/Abstract] OR healthcare[Title/Abstract] OR care[Title/Abstract] OR medical[Title/Abstract] OR clinical[Title/Abstract]) AND ("Trust"[MeSh Terms:noexp] OR trust[Title/Abstract] OR trusting[Title/Abstract] OR mistrust[Title/Abstract] OR distrust[Title/Abstract] OR antitrust[Title/Abstract] OR entrust[Title/Abstract]) AND (implement*[Title/Abstract] OR improv*[Title/Abstract] OR innovat*[Title/Abstract] OR intervent*[Title/Abstract]) AND "english"[Language] | | | | | | | | | | | | | | | | | | | |
|  |  | |  | | | | |  | | | | | | | |  | | |  | |  |  |
|  |  | | | | | |  | | |  | | | |  |  | |  |  |  |  |  |  |
|  | CINAHL (Ebsco) | | (MH "Neural Networks (Computer)" OR MH "Artificial Intelligence" OR MH "Deep Learning" OR MH "Machine Learning" OR "artificial intelligence" OR "deep learning" OR "machine learning" OR "supervised machine learning") AND ("health care" OR healthcare OR clinical OR medical) AND LA English | | | | | | | | | | | | | | | | | | | |
|  |  | | (trust OR trusting OR mistrust OR distrust OR antitrust OR entrust) AND LA English | | | | | | | | | | | | | | | | | | |  |
|  |  | | ("implement*" OR "improv"* OR "innovat*" OR "intervent*") AND LA English | | | | | | | | | | | | | | | | | | |  |
|  |  | | (trust OR trusting OR mistrust OR distrust OR antitrust OR entrust) AND (MH "Neural Networks (Computer)" OR MH "Artificial Intelligence" OR MH "Deep Learning" OR MH "Machine Learning" OR "artificial intelligence" OR "deep learning" OR "machine learning" OR "supervised machine learning") AND ("health care" OR healthcare OR clinical OR medical) AND LA English | | | | | | | | | | | | | | | | | | | |
|  |  | | (trust OR trusting OR mistrust OR distrust OR antitrust OR entrust) AND (MH "Neural Networks (Computer)" OR MH "Artificial Intelligence" OR MH "Deep Learning" OR MH "Machine Learning" OR "artificial intelligence" OR "deep learning" OR "machine learning" OR "supervised machine learning") AND ("health care" OR healthcare OR clinical OR medical) AND LA English AND (implement* OR improv* OR innovat* OR intervent*) AND LA English | | | | | | | | | | | | | | | | | | | |
|  |  | |  | | | | |  | | | | | | | |  | | |  | |  |  |
|  |  | |  | | | | |  | | | | | | | |  | | |  | |  |  |
|  | PsycINFO (ProQuest) | | noft("neural networks" OR "artificial intelligence" OR "deep learning" OR "machine learning" OR "supervised machine learning") AND noft("health care" OR healthcare OR medical OR clinical) AND DTYPE("article") AND PEER(yes) AND LA(english) | | | | | | | | | | | | | | | | | | | |
|  |  | | noft(trust OR trusting OR mistrust OR distrust OR antitrust OR entrust) AND DTYPE("article") AND PEER(yes) AND LA(english) | | | | | | | | | | | | | | | | | | | |
|  |  | | noft(implement* OR improv* OR innovat* OR intervent*) AND DTYPE("article") AND PEER(yes) AND LA(english) | | | | | | | | | | | | | | | | | | | |
|  |  | | noft("neural networks" OR "artificial intelligence" OR "deep learning" OR "machine learning" OR "supervised machine learning") AND noft("health care" OR healthcare OR medical OR clinical) AND noft(trust OR trusting OR mistrust OR distrust OR antitrust OR entrust) AND DTYPE("article") AND PEER(yes) AND LA(English) | | | | | | | | | | | | | | | | | | | |
|  |  | | noft("neural networks" OR "artificial intelligence" OR "deep learning" OR "machine learning" OR "supervised machine learning") AND noft("health care" OR healthcare OR medical OR clinical) AND noft(trust OR trusting OR mistrust OR distrust OR antitrust OR entrust) AND noft(implement* OR improv* OR innovat* OR intervent*) AND DTYPE("article") AND PEER(yes) AND LA(English) | | | | | | | | | | | | | | | | | | | |
|  |  | |  | | | | |  | | | | | | | |  | | |  | |  |  |
|  |  | |  | | | | |  | | | | | | | |  | | |  | |  |  |
|  | Web of Science Core Collection | | TS=("artificial intelligence" OR "deep learning" OR "neural networks" OR "machine learning" OR "supervised machine learning") AND TS=("health care" OR healthcare OR clinical OR medical) AND LA=(English) AND DT=(Article) | | | | | | | | | | | | | | | | | | | |
|  |  | | TS=(trust OR trusting OR mistrust OR distrust OR antitrust OR entrust) AND LA=(English) AND DT=(Article) | | | | | | | | | | | | | | | | | | | |
|  |  | | TS=(implement* OR improv* OR innovat* OR intervent*) AND LA=(English) AND DT=(Article) | | | | | | | | | | | | | | | | | | | |
|  |  | | TS=(trust OR trusting OR mistrust OR distrust OR antitrust OR entrust) AND TS=("artificial intelligence" OR "deep learning" OR "neural networks" OR "machine learning" OR "supervised machine learning") AND TS=("health care" OR healthcare OR clinical or medical) AND LA=(English) AND DT=(Article) | | | | | | | | | | | | | | | | | | | |
|  |  | | TS=(trust OR trusting OR mistrust OR distrust OR antitrust OR entrust) AND TS=("artificial intelligence" OR "deep learning" OR "neural networks" OR "machine learning" OR "supervised machine learning") AND TS=("health care" OR healthcare OR clinical or medical) AND TS=(implement* OR improv* OR innovat* OR intervent*) AND LA=(English) AND DT=(Article) | | | | | | | | | | | | | | | | | | | |
|  |  | |  | | | | |  | | | | | | | |  | | |  | |  |  |
|  |  | |  | | | | |  | | | | | | | |  | | |  | |  |  |
|  | Scopus | | TITLE-ABS-KEY("artificial intelligence" OR "deep learning" OR "neural networks" OR "machine learning" OR "supervised machine learning") AND TITLE-ABS-KEY("health care" OR healthcare OR clinical OR medical) AND DOCTYPE(ar) AND LANGUAGE(english) | | | | | | | | | | | | | | | | | | | |
|  |  | | TITLE-ABS-KEY(trust OR trusting OR mistrust OR distrust OR antitrust OR entrust) AND DOCTYPE(ar) AND LANGUAGE(english) | | | | | | | | | | | | | | | | | | | |
|  |  | | TITLE-ABS-KEY(implement* OR improv* OR innovat* OR intervent*) AND DOCTYPE(ar) AND LANGUAGE(english) | | | | | | | | | | | | | | | | | | | |
|  |  | | (TITLE-ABS-KEY (trust OR trusting OR mistrust OR distrust OR antitrust OR entrust)) AND (TITLE-ABS-KEY("artificial intelligence" OR "deep learning" OR "neural networks" OR "machine learning" OR "supervised machine learning")) AND (TITLE-ABS-KEY("health care" OR healthcare OR clinical OR medical)) AND DOCTYPE(ar) AND LANGUAGE(english) | | | | | | | | | | | | | | | | | | | |
|  |  | | (TITLE-ABS-KEY (trust OR trusting OR mistrust OR distrust OR antitrust OR entrust)) AND (TITLE-ABS-KEY("artificial intelligence" OR "deep learning" OR "neural networks" OR "machine learning" OR "supervised machine learning")) AND (TITLE-ABS-KEY("health care" OR healthcare OR clinical OR medical)) AND (TITLE-ABS-KEY(implement* OR improv* OR innovat* OR intervent*)) AND DOCTYPE(ar) AND LANGUAGE(english) | | | | | | | | | | | | | | | | | | | |
|  |  | |  | | | | |  | | | | | | | |  | | |  | |  |  |
